# PRGdb 4.0: an updated database dedicated to genes involved in plant disease resistance process

**DOI:** 10.1093/nar/gkab1087

**Published:** 2021-11-24

**Authors:** Joan Calle García, Anna Guadagno, Andreu Paytuvi-Gallart, Alfonso Saera-Vila, Ciro Gianmaria Amoroso, Daniela D’Esposito, Giuseppe Andolfo, Riccardo Aiese Cigliano, Walter Sanseverino, Maria Raffaella Ercolano

**Affiliations:** Sequentia Biotech SL, Calle Comte D’Urgell 240, 08036 Barcelona, Spain; Dipartimento di Agraria, Università di Napoli ‘Federico II’, Via Università 100, 80055 Portici, Italy; Sequentia Biotech SL, Calle Comte D’Urgell 240, 08036 Barcelona, Spain; Sequentia Biotech SL, Calle Comte D’Urgell 240, 08036 Barcelona, Spain; Dipartimento di Agraria, Università di Napoli ‘Federico II’, Via Università 100, 80055 Portici, Italy; Dipartimento di Agraria, Università di Napoli ‘Federico II’, Via Università 100, 80055 Portici, Italy; Dipartimento di Agraria, Università di Napoli ‘Federico II’, Via Università 100, 80055 Portici, Italy; Sequentia Biotech SL, Calle Comte D’Urgell 240, 08036 Barcelona, Spain; Sequentia Biotech SL, Calle Comte D’Urgell 240, 08036 Barcelona, Spain; Dipartimento di Agraria, Università di Napoli ‘Federico II’, Via Università 100, 80055 Portici, Italy

## Abstract

The Plant Resistance Genes database (PRGdb; http://prgdb.org/prgdb4/) has been greatly expanded, keeping pace with the increasing amount of available knowledge and data (sequenced proteomes, cloned genes, public analysis data, etc.). The easy-to-use style of the database website has been maintained, while an updated prediction tool, more data and a new section have been added. This new section will contain plant resistance transcriptomic experiments, providing additional easy-to-access experimental information. DRAGO3, the tool for automatic annotation and prediction of plant resistance genes behind PRGdb, has been improved in both accuracy and sensitivity, leading to more reliable predictions. PRGdb offers 199 reference resistance genes and 586.652 putative resistance genes from 182 sequenced proteomes. Compared to the previous release, PRGdb 4.0 has increased the number of reference resistance genes from 153 to 199, the number of putative resistance genes from 177K from 76 proteomes to 586K from 182 sequenced proteomes. A new section has been created that collects plant-pathogen transcriptomic data for five species of agricultural interest. Thereby, with these improvements and data expansions, PRGdb 4.0 aims to serve as a reference to the plant scientific community and breeders worldwide, helping to further study plant resistance mechanisms that contribute to fighting pathogens.

## INTRODUCTION

Green plants are ubiquitous in almost every ecosystem in the world, being the fundamental source of food and of a wide range of products (1). Nowadays, near 40% of worldwide crop production is lost due to pests and diseases (2). For this reason, plant breeders and researchers put great efforts in searching for genes involved in plant disease resistance mechanisms. Plants have developed through evolution the ability to recognize potential pathogens and predators and activate defense mechanisms to fight them (3). The activation of these mechanisms is based on specific receptors encoded by the so-called pathogen recognition genes (PRGs).

Even though different architectures can be found among PRGs, the leucine-rich repeat (LRR) domain must be highlighted for its ubiquity. This domain is present in the pattern recognition receptors (PRRs) that, being transmembrane proteins, recognize external signals and lead to the first layer of inducible defenses (also called PAMP-triggered immunity or PTI) (4). These can be divided in two main groups: RLP, that only contain LRR and, of course, transmembrane domain (TM) and RLKs, which in addition contain kinase (KIN) domain (5). Furthermore, the LRR domain is present in NLR proteins, which also contain a nucleotide-binding site (NBS) domain. These receptors are intracellular and lead to a more robust immunity (effector-triggered immunity or ETI) (6), and also can be further divided in two main groups: TNL, which in addition contain a Toll-interleukin 1 receptor (TIR) domain, and CNL, carrying an additional coiled-coil (CC) domain (7). However, there are receptors with other domains instead of LRR. For instance, there are many important PRRs for fungal pathogens that contain Lysin motif (LYSM) instead of LRR, like LYK and LYP proteins, which have a similar architecture to RLK and RLP receptors respectively, but with LYSM instead of LRR domains. Some others contain lectin-like motifs (LECM) instead of LRR, like LECRK proteins, which have similar architecture to RLK and LYK proteins (6).

Important, knowledge about plant-pathogen interactions was gained by working on individual genes. However, disease response is modulated by highly connected gene networks as well as by a cross talk between various processes and pathways. Until this date, numerous transcriptome studies have been carried out in the area of plant-pathogen interactions, establishing transcriptomics as a suitable platform to elucidate the complexity of the molecular mechanism of such interactions (8–12). Despite the advances in the omics and bioinformatics fields, data exploratory analysis is still a tedious task and the use of bioinformatics tools to study PRGs remains challenging for a significant part of the scientific community. PRGdb (13) was developed to fill this gap and, with the updated version presented here, we believe it could serve as reference to study genes involved in plant disease resistance process PRGs.

In this work, we present version 4.0 of PRGdb. PRGs classes have been expanded to 7, including LYK, LYP and LECRK receptors. The number of references PRGs has increased to 199 and the number of analyzed proteomes to 182. A new section was created that integrates public transcriptome data from studies focused on plant-pathogen interaction for five species of agricultural interest. Our annotation tool, Disease Resistance Analysis and Gene Orthology (DRAGO), has also been improved to render provide more accurate and sensitive annotations of any given DNA or amino acid (AA) sequence. All this information and other features can be browsed in our user-friendly website: http://prgdb.org/prgdb4/.

## MATERIALS AND METHODS

### New reference PRGs, classes and proteomes

An extensive bibliographic search was conducted to retrieve new cloned resistance genes from 1 August 2016 (last update of PRGdb 3.0) (14) to 1 June 2021. The aim of the search was expanded beyond LRR-containing receptors, encountering cloned genes for LYK, LYP and LECRK proteins. To test whether they were underrepresented in PRGdb 3.0, they were run through the old DRAGO2 tool. As expected, it was not able to identify these new classes. New reference PRGs were established after confirmation of their domain composition using public prediction tools: InterProScan (15), Pfam (16), CDD (17), Smart (18) and Prosite (19). A total of 51 proteins (4 RLP, 6 RLK, 6 LYP, 14 LYK and 21 LECRK) have been included as reference PRGs in PRGdb 4.0 (Supplementary Table S1).

Phytozome V13 (18,20) and Ensembl Plants release 51 (21) public databases were explored to upload any newly released proteomes since the last PRGdb 3.0 update in August 2016.

### HMM construction

The AA sequences of reference genes were separated in the seven classes of resistance genes included in PRGdb 4.0 (CNL, TNL, RLK, RLP, LYK, LYP and LECRK). Afterwards, a multiple sequence alignment (MSA) was constructed for each class using MEGA X (MUSCLE algorithm, default parameters) (22,23). Next, the MSAs were used to build Hidden Markov Models (HMMs) as described for PRGdb 3.0 (14). HMMs of each domain were built from the MSA of each class separately, with the exception of the domains LYSM (LYK and LYP proteins were also grouped together to produce additional and more robust HMMs for LYSM) and LECM (additional HMMs were also built for two subgroups within LECRK: one containing domains legume-LECM domains and another bulb-type LECM domains). Resistance domains were located within the MSA using InterProScan (SMART, Pfam, CDD and Prosite tools were activated) and MEGA software was used to visualize the MSAs and determine HMMs domain of origin.

HMMs were further filtered with *hmmsearch* (default parameters; *hmmer* tool; http://hmmer.org/) against the initial FASTA files to test whether they were indeed useful for resistance domain prediction.

### DRAGO3 pipeline

The core of DRAGO3 remained the same as the previous version DRAGO2, computing the alignment score of the different hits based on a BLOSUM62 matrix. However, HMMs were updated (except for CNL and TNL classes) and three new protein classes were added: LYK, LYP and LECRK, with all the other non-canonical domain combinations. Minimum score thresholds of DRAGO3 were defined like in the previous version PRGdb 3.0 (14), and CC domains and TM domains were equally predicted by using COILS version 2.2 (24) and TMHMM 2.0c (25) softwares, respectively.

### Validation analyses

To evaluate the performance of the new DRAGO3 tool, Araport 11 annotation of the *Arabidopsis thaliana* proteome was analyzed with both DRAGO2 and DRAGO3. The obtained putative resistance genes were then analyzed with command line InterProScan version 5.52-86.0, using Pfam 33.1 and COILS version 2.2 with the other parameters set to default, as a ground truth and compared to DRAGO results according to two criteria: (i) criterion 1 comprised proteins which have been identically predicted by DRAGO and InterProScan, (ii) criterion 2 considered proteins that have been predicted equally or better by DRAGO.

Two different validation sets, one coming from the bibliographic search (12 proteins, Supplementary Table S2) and another one from the unpublished reference database RefPlantNLR (415 proteins) (preprint available here: https://doi.org/10.1101/2020.07.08.193961), were used to calculate various performance metrics based on the Confusion matrix elements: true positive (TP), false positive (FP), true negative (TN), false negative (FN).

### RNA-seq data

An extensive literature search was performed to find publicly available RNA-seq experiments in plant-pathogen interaction in five species of agricultural interest: *Solanum lycopersicum*, *Oryza sativa*, *Triticum aestivum*, *Vitis vinifera* and *Arabidopsis thaliana*. In these studies, the different plant species were challenged with various pathogens such as bacteria, fungi, insects and viruses. For rice, wheat, grapevine and Arabidopsis, the lists of DEGs were retrieved from the corresponding works. DEGs lists from the corresponding same study were grouped for each comparative condition. For tomato, the raw sequencing data (fastq file) were downloaded from SRA repository of NCBI (https://www.ncbi.nlm.nih.gov/sra) (26) using SRA-toolkit (http://ncbi.github.io/sra-tools/) and analyzed on the same bioinformatic pipeline using web-based A.I.R. RNAseq analysis package (platform) (Sequentia Biotech, Barcelona, Spain) (https://transcriptomics.sequentiabiotech.com/) to explore and compare the different studies. Starting from raw data the AIR platform (27) performs the automatic analysis and comparison of RNA samples including the following steps: (i) quality check, (ii) adapter removal (iii) mapping, (iii) raw count calculation, (iv) DEG analysis. Tomato genome version SL3.0 ITAG 3.0 was used for the mapping.

### Relational database

The PRG data was imported into a MySQL (5.5) based relational database hosted in an Ubuntu server (14.04). The web application was developed using NodeJS technology with the ExpressJS web development framework in the back-end and HTML5, CSS3, JavaScript technologies in the front-end, besides importing libraries and frameworks such as Bootstrap or JQuery. PRGdb 4.0 is freely accessible through a web interface at the following address: http://www.prgdb.org.

## RESULTS

### DRAGO3 improvement

Initially, 209 HMMs were built from the best conserved regions extracted from the MSA of each resistance class. These HMMs were further filtered with the following criteria: (i) HMMs belonging to non-relevant regions (i.e. TM domains, which are analyzed with TMHMM tool, not with DRAGO3 HMMs); (ii) HMMs incapable of recognizing the proteins used to construct them and (iii) new HMMs were compared against DRAGO2 HMMs and those with higher performance were retained. A total of 109 HMMs were finally retained, almost doubling the previous version, DRAGO2.

### DRAGO3 validation

The analyses were carried out for all resistance domains in DRAGO3 (TIR, NBS, LRR, LYSM and LECM) except for CC and TM, since they were identified using external softwares. For the shared classes with DRAGO2 (TNL, CNL, RLK and RLP), the percentage of proteins that fulfill criterion 1 was higher in DRAGO3 for all classes (Supplementary Figure S1). For criterion 2, DRAGO3 results also were equal or higher than DRAGO2 for all classes, even though these differences were minimal (Supplementary Figure S1).

Since InterProScan is also a prediction tool, DRAGO3 was validated over two sets of reference resistance genes: (i) a set from the bibliographic search and (ii) a set from RefPlantNLR unpublished database. They were also used to extend the performance comparison with DRAGO2. Again, only the resistance domains depending on DRAGO HMMs were considered. In both validation sets, all the metrics showed overall higher performance for DRAGO3. For our validation set, DRAGO3 showed a slightly better performance. From RefPlantNLR validation, a tradeoff was observed between accuracy and sensitivity (increased from DRAGO2) and precision and specificity, which was found to be generally positive for DRAGO3 as reflected by the increase in the accuracy and *F*-score (Supplementary Figure S2), which indicates the balance between precision and sensitivity. Thus, the addition of new reference proteins for almost all classes, allowed the construction of more robust HMMs. For CNL and TNL classes though, HMMs remain the same from PRGdb 3.0.

DRAGO3 outperforms the previous version both in accuracy and sensitivity with both validation sets. It must be noted that RefPlantNLR only contains NLR proteins. Therefore, even though they carry most of the resistance domains in PRGdb 4.0, performance on the prediction of some important domains like LYSM or LECM could not be tested against any gold standard due to the reduced number of cloned genes of LYK, LYP and LECRK classes.

### New data and annotations

With the addition of three new resistance classes LYK, LECRK and LYP, PRGdb 4.0 now includes seven canonical classes of resistance proteins in plants and it is able to predict domain combinations that go beyond these well-established classes. Fifty one new proteins (4 RLP, 6 RLK, 6 LYP, 14 LYK and 21 LECRK) have been added as reference PRGs (Supplementary Table S1), leading to a total of 199 reference resistance genes now included in PRGdb 4.0. Also 182 plant proteomes were analyzed with DRAGO3 (Supplementary Table S3) and their putative resistance genes incorporated in PRGdb 4.0. Following NAR guidelines, the Sequentia in-house proteomes present in PRGdb 3.0 were removed and only leave publicly available proteomes were kept in PRGdb 4.0. Of the 182 proteomes, 39 remain unaltered from PRGdb 3.0, 33 have been updated and 110 new proteomes have been added. A total of 586 652 putative resistance genes have been predicted from these 182 proteomes. As in the previous PRGdb 3.0 release, RLK and RLP continue to be the most abundant classes with 192 544 and 150 222 total putative proteins each. Resistance classes LECRK, LYP and LYK are the least abundant with 24 959, 33 293 and 47 035 total putative proteins, respectively. A summary for all the improvements in PRGdb4.0 in comparison to PRGdb3.0 can be found in Supplementary Table S4.

### RNA-seq data integration

**Figure 1.**
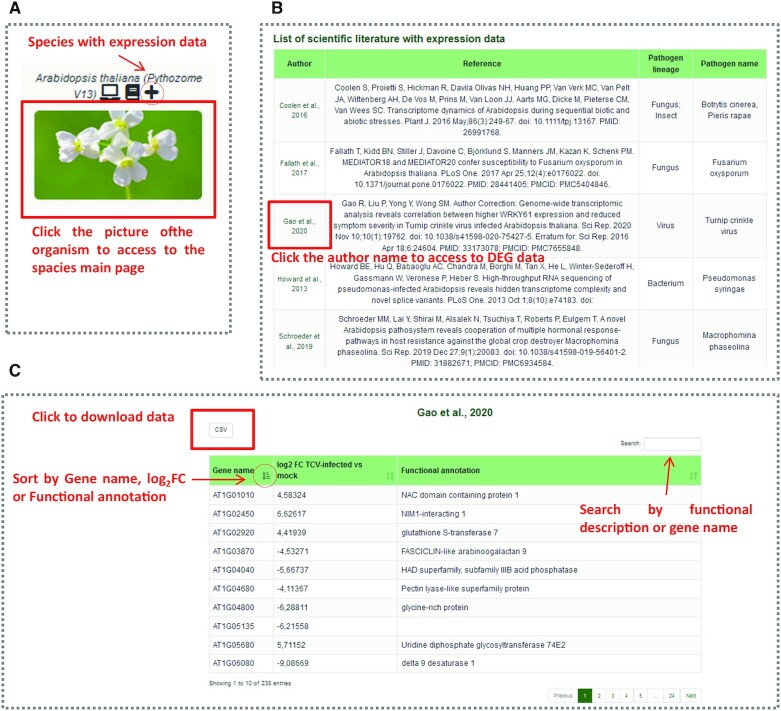
Screenshot of the new section for plant−pathogen differential expression analyses.

The extensive literature search regarding plant-pathogen transcriptome analyses yielded a total of 35 RNA-seq studies, nine for *A. thaliana*, nine for *V. vinifera*, eight for *S. lycopersicum*, five for *O. sativa* and four for *T. aestivum*. Table 1 summarizes RNA-seq studies collected, the plant species and the pathogen they interact. The list of differentially expressed genes (DEGs) under given conditions were obtained for all of them and this data has been incorporated into a new section of the PRGdb 4.0, called ‘Scientific literature with expression data’. This section is easy to access through the page dedicated to each of the five species with expression analyses (Figure 1A). In the main page available experiments for a particular species are displayed (Figure 1B). Selecting one of them will take the user to the DEG matrix (Figure 1C). The user will be able explore the results of differential expressed analysis of the various studies. This information is displayed as a DEG matrices of up- and down-regulated genes, reporting gene ID, log_2_ fold change for the comparison of interest of each study and the gene functional annotation. The user can download the data in csv format, can sort the data by gene ID, log FC and functional description. This addition will provide an experimental perspective on potential genes of interest. Such special section can be used to cross-check activated/repressed genes during different plant stresses. Here we illustrated three usage cases to identify relevant genes for genetic engineering projects, functional characterization studies and/or breeding programs in *A. thaliana* and *S. lycopersicum* datasets:

- Identification of most relevant plant species stress-related DEGs in response to a given pathogen;
- Intraspecific approach (a single gene, DEG expressed in response to more stress within the same species);
- Interspecific approach (orthologous genes, DEGs expressed in different species challenged by the same stress);
- The list of genes resulting from *A. thaliana* infected with *Verticillium* shows 11.319 DEGs at different time points (29). To identify genes expressed during early plant response against the fungus, we choose to select four moments: 0hpi, 0.25hpi, 0.5hpi and 1hpi. As suggested by Scholz (63) the fungus invades the plant tissue but does not reach the vascular system within the first 24 h of co-culture, so this timeframe is perfect to identify genes differentially regulated during early infection phases. Genes were sorted based on their Log FC (from highest to lowest to know the most activated, or viceversa). Arabidopsis AT1G01260 resulted down regulated at 0hpi (Log FC: −1.62) while its expression profile drastically changes after 0.25, 0.5 and 1hpi (Log FC: 13.04, 11.49 and 11.27, respectively). AT1G01260 (bHLH13) is a transcriptional factor that negatively regulate jasmonate responses, interacting with JAZ proteins. As reported by several studies, Jasmonates regulate plant growth and defence responses (64,65). In Arabidopsis, bHLH13 overexpression affects resistance against the necrotrophic fungi *Botrytis cinerea* and the insect *Spodoptera exigua* (66). *Verticillium dahile* is a chemibiotrophic fungi and AT1G01260 could be selected as target to test its role in plant response to *Verticillium* infection.
- Comparing two or more list of DEGs within the same species could help to have an overview on the expression profile of a particular gene under different stresses. For example, AT1G01260 (bHLH13) appears up regulated during *Botritis cinerea* and *Pieris rapae* attacks in Coolen (34) dataset and also during the fungus *Sclerotinia sclerotiorum* infections in Sucher (30). These insights could be useful to plane further genetic engineering experiments to better understand gene function, which could affect resistance or other related traits.
- Interspecific approach could be conducted comparing two different species affected by the same pathogen. In our example, we compare Arabidopsis and tomato infected with *Sclerotinia sclerotiorum*. A short list among the up and down regulated genes in both experiments was analyzed. We found that Arabidopsis AT2G41820 (PXC3) orthologous of tomato Solyc04g015600 (LRR family protein), resulted down regulated in both dataset (Log FC −4.78 and −1.79, respectively).

**Table 1.** Plant-pathogen transcriptomic data added to PRGdb RNAseq section

Plant species	Pathogen	Pathogen name	Reference
**Arabidopsis**			
*Arabidopsis thaliana*	Bacteria	*Pseudomonas syringae*	(28)
*Arabidopsis thaliana*	Fungus	*Verticillium dahliae*	(29)
*Arabidopsis thaliana*		*Sclerotinia sclerotiorum*	(30)
*Arabidopsis thaliana*		*Macrophomina phaseolina*	(31)
*Arabidopsis thaliana*		*Fusarium oxysporum*	(32)
*Arabidopsis thaliana*		*Fusarium oxysporum*	(33)
*Arabidopsis thaliana*	Fungus; insect	*Botrytis cinerea, Pieris rapae*	(34)
*Arabidopsis thaliana*	Virus	*Turnip crinkle virus*	(35)
*Arabidopsis thaliana*	Aphid	*Myzus persicae (GPA)*	(36)
**Grape wine**			
*V. vinifera* spp. *Sativa, V. vinifera* spp. *Sylvestris*	Fungus	*Erysiphe necator*	(37)
*Vitis pseudoreticulata*		*Erysiphe necator*	(38)
*Vitis. vinifera*		*Botrytis cinerea*	(39)
*Vitis vinifera*		*Botrytis cinerea*	(40)
*Vitis vinifera*		*Neofusicoccum parvum*	(41)
*Vitis vinifera*		*Lasiodiplodia theobromae*	(42)
*Vitis vinifera*		*Erysiphe necator*	(43)
*Vitis vinifera*		*Plasmopara viticola*	(44)
*Vitis vinifera*	Phytoplasma; insect	*Flavescence dorèe; Scaphoideus titanus*	(45)
**Tomato**			
*Solanum lycopersicum*	Bacteria	*Pseudomonas syringae* pv. tomato DC3000	(46)
*Solanum lycopersicum* and *Solanum Pimpinellifolium*		*Ralstonia solanacearum*	(47)
*Solanum lycopersicum*	Fungus	*Phytophthora infestans*	(48)
*Solanum lycopersicum*		*Cladosporium fulvum*	(49)
*Solanum lycopersicum*		Sclerotinia sclerotium	(50)
*Solanum lycopersicum*	Virus	*Southen Tomato Virus*	(51)
*Solanum lycopersicum*		*Tomato spotted wilt tospovirus*	(52)
*Solanum lycopersicum*	Pest	*Tuta absoluta*	(53)
**Rice**			
*Oryza sativa* L. cv. *Nipponbare*	Bacteria	*Xanthomonas oryzae* pv. *oryzae*	(54)
*Oryza sativa* L. and *Oryza meyeriana L*.		*Xanthomonas oryzae* pv. *oryzae*	(55)
*Oryza sativa* L. spp. *Japonica*	Fungus	*Magnaporthe oryzae*	(56)
*Oryza sativa*		*Fusarium fujikuroi*	(57)
*Oryza sativa* L. spp. *Japonica*		*Fusarium fujikuroi*	(58)
**Wheat**			
*Triticum aestivum L*.	Fungus	*Fusarium pseudograminearum*	(59)
*Triticum aestivum L*.		*Puccinia striiformis* f. sp. *tritici*	(60)
*Triticum aestivum L*.		*Tilletia controversa Kühn*	(61)
*Triticum aestivum L*.	Bacteria	*Azospirillum brasilense*	(62)

We consider that data cross-comparison can represent a powerful tool to discover new important genes involved in plant−pathogen interaction. The above case are examples of potential analyses that can be carried out exploring the new expression data sections of our database.

## CONCLUSION AND PERSPECTIVES

As sequencing becomes more affordable and efficient, plant genome data availability is rapidly increasing. The extraction of meaningful results and conclusions from this data depends on data quality, and exploratory analyses are fundamental to assess this quality. Furthermore, crop research has diversified to several useful species and new resistance strategies are being studied. PRGdb4.0 is an updated database with increased utility to the plant scientific and breeding communities. It can be used to consult plant resistance genes for many plants and algae, to analyze sequences for the prediction of resistance genes and to investigate gene expression under specific plant-pathogen conditions.

The new incorporation of transcriptomic data to PRGdb 4.0 will offer researchers and breeders a more comprehensive perspective of the plant response to pathogens for most relevant plant species. Furthermore, the update of our prediction and annotation tool DRAGO3 will improve the quality of exploratory analyses. The addition of new proteomes will surely allow the study of species that was not possible to date.

PRGdb will continue to grow by incorporating new reference PRGs and genomes as they become publicly available. We will also continue to work on multi-omics data integration in PRGdb, with the aim of becoming a reference database for plant researchers to address critical challenges in plant-pathogen interactions.

## DATA AVAILABILITY

PRGdb 4.0 is available at: http://prgdb.org/prgdb4/.

## Supplementary Material

gkab1087_Supplemental_Files
