# Supplementary material for: PRGdb 4.0: an updated database dedicated to genes involved in plant disease resistance process
Source: Nucleic Acids Res. 2021 Nov 24;50(D1):D1483–90. doi: 10.1093/nar/gkab1087 (PMC8729912; doi:10.1093/nar/gkab1087)
Supplement: gkab1087_Supplemental_Files [file gkab1087_supplemental_files.zip › Supplementary figures.pdf]

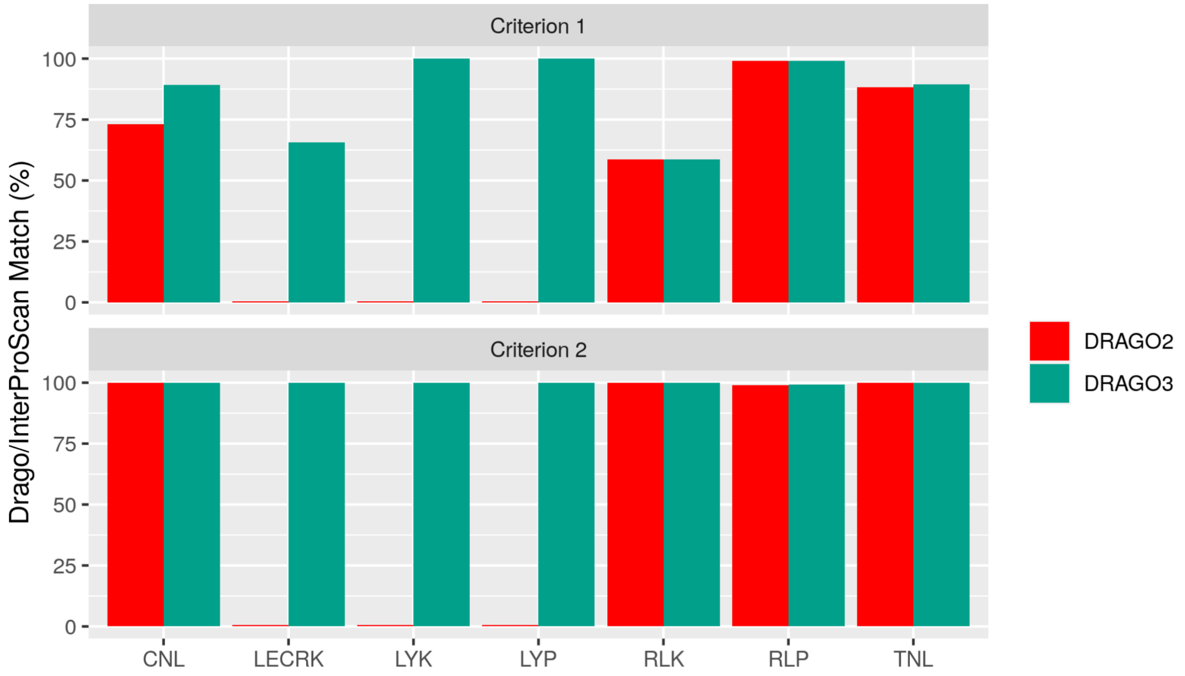

Supplementary figure S1. Performance test of DRAGO3 by comparing predictions with InterProScan results according to “criterion 1” (upper plot) and according to “criterion 2” (lower plot). DRAGO2 is red, DRAGO3 is blue.

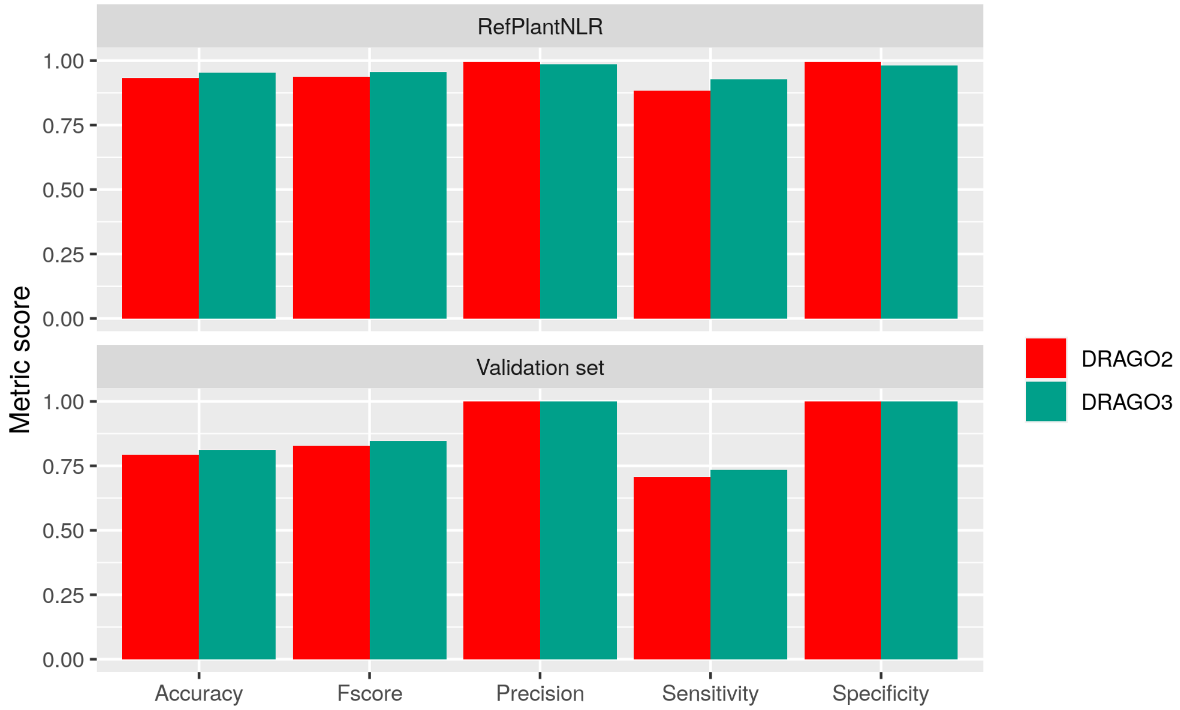

Supplementary figure S2. Performance test of DRAGO3 by comparing predictions with domain composition of two sets of reference plant NLR proteins: RefPlantNLR set from unpublished work (https://doi.org/10.1101/2020.07.08.193961) (top plot) and a validation set from bibliographic search (bottom plot). DRAGO2 is red, DRAGO3 is blue. Calculated performance metrics: Accuracy [ratio of correct predictions to the total number of inputs; (TP + TN) / (TP + FP + FN + TN)], Precision [indicates how many predictions are indeed relevant: TP / (TP + FP)], Sensitivity [addresses the ratio of relevant predictions from all possible correct predictions; TP / (TP + FN)], Specificity [measures how many negative predictions are truly negative; TN/(TN+FP)] and F-score [finds the balance between precision and sensitivity; (2 x Precision x Sensitivity) / (Precision + Sensitivity)].
